# Acute severe ulcerative colitis: using JAK-STAT inhibitors for improved clinical outcomes

**DOI:** 10.3389/fgstr.2024.1488288

**Published:** 2024-11-25

**Authors:** Shruthi Karthikeyan, Chetan Ambastha, Kian Keyashian

**Affiliations:** ^1^ School of Medicine, St. George’s University, St. George’s, Grenada; ^2^ Department of Medicine, School of Medicine, Stanford University, Palo Alto, CA, United States; ^3^ Division of Gastroenterology and Hepatology, Department of Medicine, School of Medicine, Stanford University, Palo Alto, CA, United States

**Keywords:** acute severe ulcerative colitis (ASUC), infliximab (IFX), salvage therapies, JAK inhibitors, tofacitinib, upadacitinib

## Abstract

Acute Severe Ulcerative Colitis (ASUC) is a well-known and potentially fatal disease state, characterized by symptoms of systemic toxicity including fever, severe anemia, elevated inflammatory markers, and autonomic instability. The life-threatening nature of this condition requires clinicians to make prompt diagnoses and take rapid action, either directing patients towards surgical interventions or medical management. Failure to treat ASUC may lead to toxic dilation of the colon, hemorrhage, or sepsis. Current algorithms suggest the use of intravenous (IV) corticosteroids upon diagnosis, with transition to oral corticosteroids, calcineurin inhibitors or tumor necrosis factor (TNF) inhibitors upon reduction of severe symptoms for candidates deemed to be amenable to medical management. Within these classes, TNF inhibitors such as Infliximab (IFX) have proven to be the most safe, efficacious, and tolerable for patients. While IFX has much data supporting its benefits in achieving short term remission, there are still high rates of long-term need for colectomy and failure to maintain remission. This is due to interactions between the inflamed gastrointestinal tract, the increased metabolic activity seen in ASUC, and intrinsic pharmacodynamic properties of IFX. Certain novel studies suggest that Janus Kinase (JAK-STAT) inhibitors such as Tofacitinib and Upadacitinib are potent agents to salvage clinical remission achieved by IFX, upon its failure. Here we discuss methods to optimize the dosing of IFX to maximize its efficacy, while exploring recent work done on the safety and efficacy of JAK-STAT inhibitors as a salvage therapy, therefore suggesting a novel treatment algorithm to improve clinical outcomes in medically managed ASUC patients.

## Introduction

### Acute severe ulcerative colitis

Ulcerative Colitis (UC) describes a subset of Inflammatory Bowel Disease (IBD) characterized by diffuse immune-mediated inflammation of the gastrointestinal (GI) tract usually involving the rectum and extending proximally in the colon. Chronic inflammation results in the development of ulcers, erosions, and bleeding, complicating patient outcomes. Without consistent treatment, patients often present with alternating periods of flare and clinical remission – the duration and severity of which is unpredictable. Typical flares of UC present with generalized abdominal pain, fever, nausea, tenesmus, fecal urgency, diarrhea and/or constipation with or without blood or mucus in the stool. Acute Severe Ulcerative Colitis (ASUC) is a life-threatening complication of UC, characterized by a significant exacerbation of the disease state. ASUC is diagnosed based on the Truelove and Witts criteria, which includes passing at least 6 bloody stools a day alongside at least one of the following symptoms of systemic toxicity: a temperature of over 37.8˚C, hemoglobin levels of less than 1.5g/dL, erythrocyte sedimentation rate (ESR) of more than 30mm/h and/or a pulse rate of at least 90bpm (1). Nevertheless, clinicians may diagnose patients who do not fully meet criteria based on individual presentation. Several reports indicate 10-20% of patients require colectomy upon their first episode of ASUC and patients with more than one episode of ASUC have a 30-40% risk of requiring colectomy, highlighting the significant morbidity associated with this condition (2, 3).

**Figure 1:**
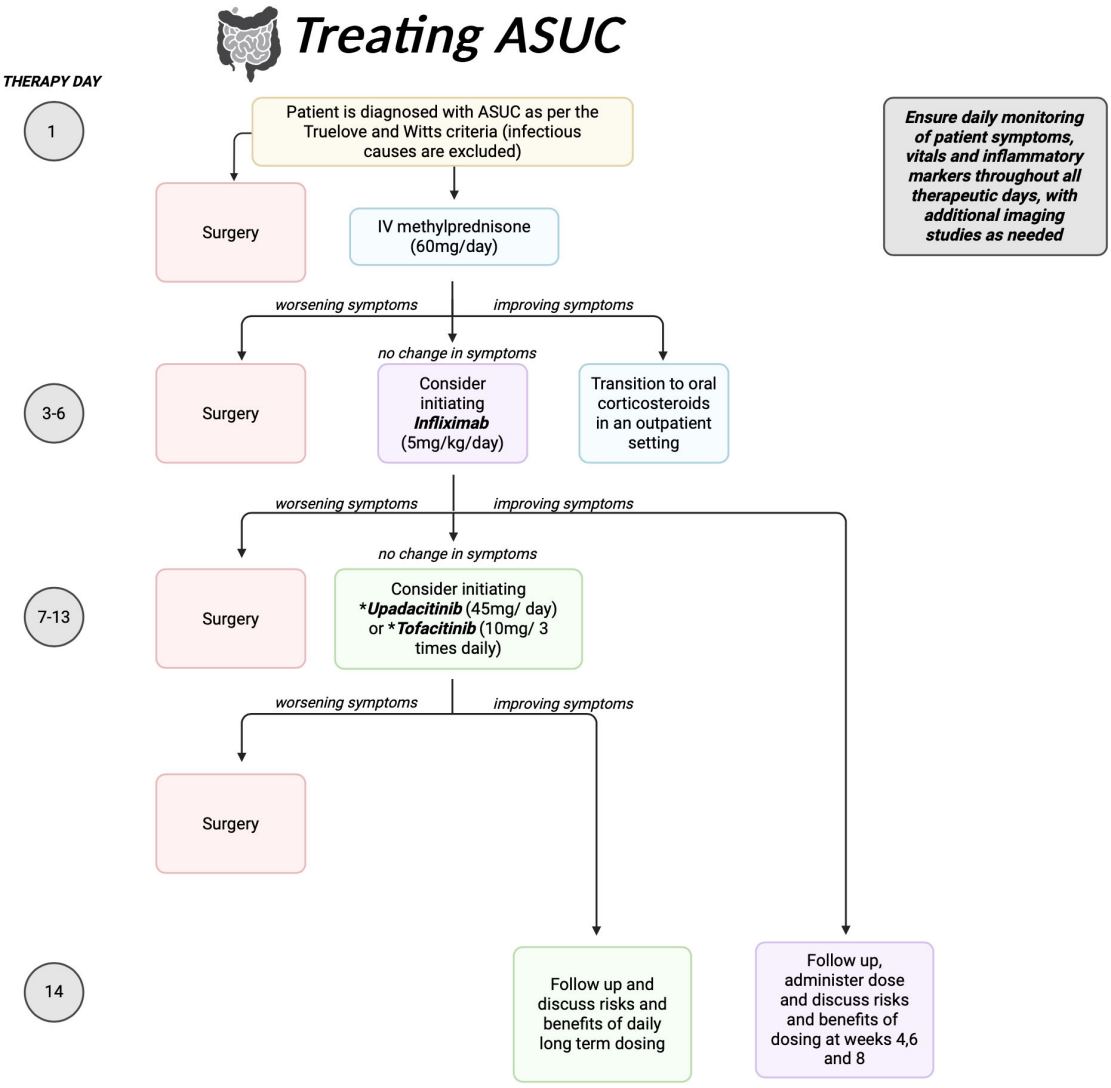
Time based algorithm for the management of Acute Severe Ulcerative Colitis.

In this review, we explore methods to optimize administration of advanced therapies in management of ASUC, including infliximab and Janus Kinase (JAK) inhibitors. Our goal is to develop an algorithm to improve clinical outcomes and reduce need for colectomy in ASUC patients (**Figure 1**).

### Initial management of ASUC

The mainstay of initial management of ASUC relies on early diagnostics – blood testing for inflammatory markers, stool testing for pathogens such as *Clostridioides difficile*, sigmoidoscopy with biopsy to assess endoscopic severity and rule out cytomegalovirus infection, and imaging if there are atypical features, such as focal severe abdominal pain. While patients with C. Difficile infection should be promptly treated with oral vancomycin or fidaxomicin (4), the American Gastroenterological Association (AGA) recommends against empiric antibiotics in patients without proven infection. Four randomized controlled trials were included in a meta-analysis which estimated there was equivalent risk of colectomy in ASUC patients administered and not administered vancomycin (RR 0.95; 95% CI, 0.55-1.64) (5–8). Cytomegalovirus infection is an additional consideration early in presentations, particularly in patients on chronic immunosuppressive therapy and unresponsive to an outpatient course of prednisone. If sigmoidoscopy showed classic deep ulceration with biopsies showing cytopathic effect from CMV, treatment could be considered – particularly if the patient is not responding to standard therapy. In such cases, intravenous (IV) ganciclovir (5mg/kg twice daily) or oral valganciclovir (900mg twice daily) could be considered for the course of 1 week (9).

Subsequent medical management of ASUC follows a time-based algorithm based on evaluation on each day of hospitalization (2). Prophylaxis for venous thromboembolism (VTE) is provided as this has shown to result in significant reduction in thromboembolism rate without significant increase in risk for major bleeding (10, 11). On the first day of hospitalization, patients are promptly evaluated for need of surgery as this early evaluation results in significantly improved outcomes (12). Patients who do not require surgery and have no evidence of infection are started on IV corticosteroids. Current studies indicate that optimal dosing of IV methylprednisone is 60mg (13); however further studies are needed comparing steroid regimens. Patients must be monitored on corticosteroids on days 2 and 3 for any change in daily stools, pain, bleeding, inflammatory markers.

Recent studies indicate that approximately one third of patients fail to respond to IV corticosteroids within 3-5 days, prompting the need for rescue therapy such as infliximab or cyclosporine (1). The Travis criteria (1996) describes a method to predict risk of corticosteroid failure in patients with ASUC, stating that patients who stool more than 8 times a day or stool over 3 times a day alongside having a C-Reactive Protein (CRP) level of over 45mg/L have an 85% likelihood of needing colectomy (13, 14). Other criteria such as the Ho, Lindgren, Seo and Jain criteria also attempt to stratify risk based on inflammatory markers and severity of colonic dilation (2, 15–18). Day 4 of hospitalization presents as a critical point of evaluation as to whether to continue therapy or move onto surgical methods. Patients with toxic dilatation, impending perforation, massive hemorrhage, or longstanding intractable colitis should be promptly moved to surgery for colectomy. In addition, patients who are unresponsive to corticosteroid therapy within 7 days of initiation are generally recommended to undergo urgent colectomy (1, 2).

### Rescue therapies for ASUC

Patients with inadequate response to IV corticosteroids are candidates for rescue therapies. Currently available therapeutic options include cyclosporine, tacrolimus, and infliximab (2) (**Table 1**). Cyclosporine is a calcineurin inhibitor, helping terminate T lymphocyte activity and subsequent cytokine gene transcription (35). Its use in the treatment of ASUC was initially reported by Lichtiger et al. (23) who conducted a randomized trial (RCT) of continuous IV cyclosporine at varying doses of 2mg/kg/day or 4mg/kg/day compared to placebo. One week after the initiation of treatment, 82% of patients on cyclosporine achieved clinical response compared to 0% in the placebo group. The efficacy of cyclosporine was confirmed in subsequent case series and RCT’s, which suggested that the use of 2mg/kg/day of medication yielded similar response to patients on 4mg/kg/day and that the target serum concentration of cyclosporine should be between 150 to 250 ng/mL (2, 24). The most commonly reported side effects with cyclosporine usage have been nephrotoxicity, seizures and anaphylaxis (2). Long-term prognosis on treatment has been mixed, with finding of approximately 33% of patients requiring colectomy at 1 year and 88% at 7 years (25).

**Table 1:** Benefit and risk considerations for different rescue and salvage therapies used in the treatment of Acute Severe Ulcerative Colitis.

Type of Therapy	Biologic	Benefit Considerations	Risk Considerations
**Rescue**	*Infliximab*	Over 50% of patients achieve clinical response by week 2 (12, 13) Significantly higher therapeutic response rate compared to those treated with either cyclosporine or tacrolimus (OR=3.15, 95% CI=2.26-4.40) (19). Significantly reduced first year colectomy rate following initiation of treatment (OR=0.46, 95% CI =0.27-0.79) (19) More readily usable as a maintenance therapeutic and has a better safety profile when compared to calcineurin inhibitors (19).	Rapidly cleared in ASUC patients (19–22). Factors contributing to increased clearance include: Low body weight Increased mucosal TNF Elevated fecal calprotectin Lactoferrin pANCA male sex
	*Cyclosporine*	One week after the initiation of treatment 82% of patients on cyclosporine achieved clinical response (23) Dosage regimes of 2mg/kg/day and 4mg/kg/day yield similar clinical results (23) Target concentration of cyclosporine is determined to be between 150 to 250 ng/mL (2, 24)	The most commonly reported side effects with cyclosporine (2): nephrotoxicity seizures anaphylaxis Approximately 33% of patients requiring colectomy at 1 year and 88% at 7 years (25)
	*Tacrolimus*	Two weeks after the initiation of treatment, 68% of those in the high trough group (10-15ng/mL), 38.1% of the low trough group (5-10ng/mL) and 10% of the placebo group achieved clinical response (26) Colectomy free rates at 1,3,6 and 12 months are 86%, 84%, 78% and 69%, respectively (27). Exhibits good oral bioavailability (2).	Short term failure is estimated to be 30% whilst long term failure is greater than 50% (2)
**Salvage**	*Cyclosporine*	Colectomy rates at 3 and 12 months were 28% and 42% respectively (28)	Delay of colectomy and additive effects of immunosuppression from both rescue and salvage therapy Increased risk of serious infection and death (28)
	*Upadacitinib*	Good safety profile compared to calcineurin inhibitors Rapid onset of action with results seen as early as 1 day (29)	Limited data surrounding efficacy and safety When co-administered with IV corticosteroids, 24% of patients necessitated or opted for colectomy within 90 days of treatment (30)
	*Tofacitinib*	Rapid results within 3 days of therapeutic administration (31) Colectomy only necessary in 16.7% of patients by day 7 and 25% of patients by month 6 (32) Persistence of remission at follow up past 180 days ranged between 68-91% and endoscopic remission was seen in 55% of patients (33) May be as effective as prednisolone for the induction of remission (34)	Risk of herpes zoster infection (33) Limited data surrounding efficacy and safety

Tacrolimus is another calcineurin inhibitor used as a rescue therapy of ASUC. Ogata et al. conducted an RCT evaluating the use of tacrolimus in steroid refractory ASUC patients. Patient were treated with an initial dose of 0.025mg/kg of tacrolimus or placebo twice daily and were then further stratified into high trough groups (10 to 15ng/mL) or low trough groups (5 to 10 ng/mL). Patients were monitored for disease activity score (DAI) at different time points throughout 10 weeks. Two weeks after the initiation of treatment, 68% of those in the high trough group, 38.1% of the low trough group, and 10% of the placebo group achieved clinical response (26). The dose-dependency of tacrolimus in ASUC has been redemonstrated in many subsequent studies (26, 36). Short-term efficacy is estimated to be 70% while long-term efficacy is greater than 50% (2). In a meta-analysis including 934 patients with severe or steroid refractory UC treated with tacrolimus, colectomy free rates at 1,3,6 and 12 months were 86%, 84%, 78% and 69%, respectively (37). Moreover, tacrolimus’ good oral bioavailability and tolerability make it more amenable than cyclosporine as a long-term treatment option (2). In a retrospective study consisting of 22 steroid refractory ASUC patients, 86.4% of the group was discharged on oral tacrolimus and colectomy free survival rates at 1,3,6 and 12 months were 90.9%, 86.4%, 77.3% and 68.2% respectively. Only 2 of these patients were unable to tolerate tacrolimus due to its side effects (27).

Infliximab (IFX) is a tumor-necrosis-factor alpha (TNF-a) inhibitor that may also be used as a rescue therapy, typically dose based on weight at 5mg/kg. Many studies have highlighted its efficacy in achieving short-term remission (2, 20). Sands et al. compared patients administered 5, 10 or 20mg/kg of IFX to patients on equal level of placebo, through an RCT. Although there were only 11 patients studied, half of the patients treated with IFX achieved clinical response by week 2, whereas all of those treated with placebo had to undergo colectomy (38). Similar results were seen in a study that randomized 45 patients with ASUC who were administered either a single dose of IFX 5mg/kg or placebo. This resulted in 66% of the placebo group undergoing colectomy after 1 month of treatment initiation versus 29% requiring colectomy in the IFX treated group at the same time point (39).

Many studies have compared the efficacy of infliximab to other rescue agents for treatment of ASUC. A meta-analysis done by Chang et al. compared infliximab and cyclosporine as rescue therapy and included 321 patients with steroid-refractory UC; there were no significant differences between IFX and cyclosporine in the reduction of colectomy rates at both 3 months (OR=0.86, 95% CI =0.31-2.41, p=0.775) and 12 months (OR=0.60, 95% CI= 0.19-1.89, p=0.381) (40). There were also no significant differences in incidence of post-operative complications (OR=1.66, 95% CI = 0.26-10.50, p=0.591) or drug reactions (OR=0.76, 95% CI=0.34-1.70, p=0.508) (40). Another meta-analysis done by Narula et al. evaluating patients with steroid refractory UC found no significant difference in 3 and 12 month colectomy rates, incidence of adverse drug events, post-operative complications or mortality between IFX treated or cyclosporine treated patients when assessing three randomized studies (41). However, when evaluating non-randomized studies, they found a significantly increased treatment response (OR= 2.96, 95% CI 2.12-4.14, x^2^ = 6.50, I^2^ = 0%) and a lower 12 month colectomy rate (OR= 0.42, 95% CI 0.22-0.83, x^2^ = 30.94, I^2^ = 71%) in patients treated with IFX compared to tacrolimus (41). Liu et al. also found similar efficacy when comparing infliximab and tacrolimus in 438 cases of steroid refractory ASUC in an RCT. Short-term clinical response rates, clinical remission rates, and 3-month colectomy rates were 72.1%, 52.4% and 10.1% respectively in IFX treated patients; similar outcomes were noted in the tacrolimus treated group (76.9%, 48.8% and 12.4%, respectively). An increased rate of adverse events were seen in patients treated with tacrolimus when compared to those treated with IFX (OR=2.16, 95% CI =1.25-3.76, p=0.006) (42).This increased rate of adverse events was influenced by one particular study which reported hypomagnesemia in the vast majority of patients receiving tacrolimus; when this study was excluded, there was no significant difference in adverse events supporting the safety and efficacy of tacrolimus in these patients.

Additional large studies indicate IFX’s potential superior placement above both tacrolimus and cyclosporine as rescue therapies, though differences in these conclusions are noted in observational versus randomized studies. Zhao et al. conducted a meta-analysis comprised of 19 studies with 1323 ASUC patients refractory to steroids (19). Patients treated with IFX had a significantly higher therapeutic response rate when compared to cyclosporine or tacrolimus (OR=3.15, 95% CI=2.26-4.40) when analyzing non-randomized studies. IFX was also associated with a significantly reduced first-year colectomy rate following initiation of treatment (OR=0.46, 95% CI =0.27-0.79); this trend continued with respect to colectomy rates at year two (OR=0.53, 95% CI=0.28-0.97) and year three (OR=0.43, 95% CI =0.24-0.75). The study did not show any significant differences in the rates of adverse events, mortality or colectomy between the groups when analyzing randomized controlled trials (19).

Regarding safety, IFX also has the benefit of being more readily usable as a maintenance therapeutic with a better safety profile when compared to calcineurin inhibitors (19). A review by Rosen et al. highlights concerns with long-term usage of IFX for ASUC, with approximately half of the patient population requiring colectomy at some point. This is theorized to be in part due to rapid clearance of the agent in ASUC patients caused by disease severity (20).

## Optimizing IFX dosing for ASUC

### Pharmacodynamics of IFX in ASUC patients

Rosen et al. proposed a theory as to why it is difficult to achieve optimal levels of anti-TNF agents in ASUC patients (43). As explained in their review article, the sponge, shark and sieve metaphor refer to patients absorbing and thereby clearing these agents, proteolytically cleaving these agents, and losing these agents through gut leakage at higher rates than in patients without ASUC. Certain studies have indicated that patients with more severe of UC have a proportional increase in expression of TNF in their mucosa, macrophages and lymphocytes, resulting in increased binding and clearance of anti-TNF agents (21). Olsen et al. suggested that the clinical outcome of IFX therapy is inversely correlated with the level of gene expression of TNF-alpha in the colorectal mucosa of patients, noting 82% of patients with low pre-treatment mucosal TNF expression achieved mucosal healing compared to 42% of patients with high pre-treatment mucosal TNF (22).

Once administered anti-TNF agents bind to mucosal TNF receptors and form complexes which are subject to Fc-receptor-mediated endocytosis and proteolytic degradation by the reticuloendothelial system (44). Immune-mediated inflammation appears to upregulate reticuloendothelial activity, which is then compounded by increased mucosal TNF expression lending to increased break down of anti-TNF agents in ASUC patients (43). The final component of the theory as to why ASUC patients metabolize anti-TNF agents more rapidly lies in increased protein losses seen in the diseased colon. Increased “leakiness” of the GI tract caused by inflammation in IBD and especially ASUC lends to albumin and immunoglobulin losses. This is confirmed by Brandse et al. who measured IFX levels in the stool of treated patients, revealing that stool level of IFX was inversely proportional to attainment of endoscopic remission (45).

Based on these observations and potential theoretic mechanisms, it may be possible to identify factors that may make patients more prone to anti-TNF failure. One factor would be low serum albumin, which points towards a damaged and thus highly permeable GI tract. A second factor would be increased mucosal TNF levels. Other studies have indicated low body weight, elevated fecal calprotectin, lactoferrin, Mayo score, presence of pANCA, and male sex to be negative prognostic factors with regards to anti-TNF therapy (43, 46, 47).

Integrating this data, the currently underway TITRATE trial by D’Haens et al. aim to investigate the development and use of a pharmacokinetics driven dashboard in order to develop personalized IFX dosing. The group aims to develop a protocol to guide proactive adjustments in IFX dosing in point-of-care settings based on individual patient pharmacokinetic characteristics, as opposed to current time consuming enzyme linked immunosorbent assay (ELISA) methods to determine levels of and appropriate doses of IFX for ASUC patients (48). Similar development of dashboards have previously been done in CD and UC patients with varying levels of disease activity, through the analysis of individual patient weight, albumin parameters and other factors to predict serum infliximab concentration for particular IFX doses (49, 50).

### Standard IFX dosing in ASUC patients

Current dosing guidelines for IFX follow a standard induction dose of 5mg/kg infused at weeks 0,2,6 and every 8 weeks thereafter (51). While IFX is effective in the treatment of ASUC and yields similar results to other calcineurin inhibitors, patients treated with this therapy remain at high risk of requiring colectomy long term. Certain groups propose that this may be due to inadequate or suboptimal dosing as a result of the pathophysiology of ASUC and its effects on drug metabolism.

Numerous observational studies in IBD have shown an exposure-response relationship for biologic therapies, with more studies in infliximab and adalimumab than other agents (52). Seow et al. performed an RCT on 115 patients with UC who were administered a three-dose series of IFX induction and maintenance therapy, who were then followed for rates of remission, colectomy, presence of antibodies to IFX and trough level of IFX (52). At week 10 and 54, 32% and 37% of patients on IFX achieved remission, whereas 40% of patients required colectomy as some point. 39% of the patients had detectable trough levels of IFX; amongst the patients who had undetectable trough levels, 41% had antibodies to IFX and 20% did not. The presence of antibodies did not significantly alter the achievement of remission, endoscopic improvement or need for colectomy. However, patients with detectable levels of IFX in their serum had significantly higher rates of remission when compared to those who did not (69% vs. 15%, p<0.001). Similar results were seen in the attainment of endoscopic improvement (76% vs. 28%, p<0.001). Moreover, patients with undetectable serum levels of IFX had a significantly increased risk of needing colectomy (55% vs. 7%, OR= 9.3, 95% CI 2.9-29.9, p<0.001) (52). Adedokun et al. assessed similar questions through analysis of the Active Ulcerative Colitis Trials (ACT-1 and ACT-2), proposing an optimal trough level of IFX for positive patient outcomes in ASUC (53). When comparing serum concentrations of IFX in 728 patients with ASUC at weeks 8, 30 and 54, patients with higher serum levels were more likely to attain clinical response, mucosal healing, and clinical remission. Their study indicates that approximately 41ug/mL of IFX at week 8 and 3.7ug/mL at steady state were associated with optimal patient outcomes. The study also pointed towards a relationship between lower albumin levels and higher risk of IFX failure (53).

### Accelerated IFX dosing for ASUC

While some studies indicate that accelerating the standard regime for IFX treatment in ASUC patients has no bearing on outcomes (54), many of the studies highlight benefits of accelerated dosing in selected populations. Choy et al. recently delved into identifying an optimal dosing strategy of IFX in ASUC patients through an open-label RCT conducted at 13 Australian centers (55). 138 patients were randomized, with 46 patients receiving an induction IFX dose of 10mg/kg and 92 patients receiving 5mg/kg. All patients in the increased dose group received an additional dose at day 7 or at time of loss of response. Patients in the lower dose group were re-randomized to either receive 5mg/kg IFX at week 0,2 and 6 with an additional dose at day 7 or no response (SI) or receive 5mg/kg at week 0,1 and 3 with increase of dose to 10mg/kg at day 7 or no response (AI). Results revealed that clinical response was achieved in 65% of patients administered 10mg/kg IFX, and 61% of those administered 5g/kg IFX. 2 patients who were administered 10mg/kg IFX had to undergo colectomy at day 7, as opposed to 0 patients in the 5mg/kg group, suggesting an optimal dose of IFX to maximize safety without greatly compromising efficacy. Differences between rates of clinical, biochemical, endoscopic, and steroid free remission were not significantly different between the SI and AI groups; however the AI group did achieve remission sooner. The group did also see an albumin associated decrease in achievement of remission. In the 5mg/kg group, clinical response rate was lower in those with albumin levels <25g/L when compared to those ≥25g/L (47% vs. 68%, p=0.07) whereas this association was not evident in the 10mg/kg group (64% vs. 66%, p>0.99) (55). This work suggests that while there is minimal difference in patients receiving 10mg/kg IFX vs. 5mg/kg IFX with regards to clinical outcomes, accelerated or intensified induction may help patients achieve remission sooner, especially those with low albumin levels. The currently ongoing PREDICT trials aim to elucidate the benefits of accelerated dosing further (55, 56).

A retrospective analysis by Hefarth et al. also highlights the short-term benefits of 5mg/kg IFX dosing in ASUC (57). This group compared 15 patients receiving accelerated 5mg/kg induction IFX over 2 weeks followed by q8 maintenance doses to 35 patients receiving standard 5mg/kg induction IFX over 6 weeks followed by q8 weekly maintenance. 6.7% of the accelerated dose group compared to 40% of the standard dose group presented with need for colectomy at the 3 month follow up, supporting the benefit of accelerated dosing. This difference, however, was not present at 6 month and 12 month time points (57). These results were echoed in a review analyzing 76 studies, ultimately showing that IFX dose intensification was beneficial to 50% of ASUC patients and 1-2 additional doses of IFX within the first 3 weeks of treatment reduced early colectomy rates by up to 80% (58).

## Salvage strategies for subclinical effects with IFX

Given the currently landscape of therapeutics and early approval of infliximab, a number of patients hospitalizes with ASUC have already trialed and failed infliximab. In addition, IFX has limitations with regards to maintaining remission and reducing colectomy rates in some patients. Thus, recent studies have aimed at finding effective salvage therapies in cases of IFX failure, with most work point towards the use of cyclosporine (**Table 1**).

### Cyclosporine

Evidence supporting the use of calcineurin inhibitors as a salvage therapy for IFX failure is limited by the small number of clinical studies, all of which include small numbers of patients. The largest study evaluating this practice was done by Weisshof et al., who conducted a retrospective analysis evaluating 40 patients at a tertiary center with steroid refractory ASUC. This group was the first to report IFX levels in patient blood prior to cyclosporine administration (59). These patients were treated with IV cyclosporine after failing IV steroids and IFX within the previous 2 months, and subsequently followed for 13 months. Evaluation of patients at 1 month, 3 months and 1 year revealed colectomy free survival in 65%, 59.4% and 41.8% of patients respectively. Several additional studies have examined the use of IFX and cyclosporine as rescue therapies for previous failure of the other agent. A review performed by Gisbert et al. evaluating different agents for steroid refractory ASUC highlights concerns regarding use of IFX including colectomy rates and cost of therapy (60). This group analyzed 23 studies comprised of 340 patients and revealed that rescue therapy avoided colectomy in 53% of patients (95% CI 47%-58%). A meta-analysis done in 2015 analyzing 10 studies, revealed that after sequential treatment with cyclosporine following IFX treatment, 39% of patients achieved short-term remission. Colectomy rates at 3 and 12 months were 28% and 42% respectively (28). Adverse events were seen in 23% (95% CI 17.7%-28.3%) of patients with serious infection occurring in 6.7% (95% CI 3.6%-9.8%) and death in 1% (95% CI 0%-2.1%) (28).

An important factor to consider before the initiation of cyclosporine as a salvage therapy for IFX failure is safety. Gisbert et al. calculate a 26% rate of adverse events and a 0.88% rate of mortality when including 14 studies using cyclosporine as rescue therapy for infliximab or vice versa (61). Similarly, another meta-analysis calculated a 23% rate of adverse events, of which there were serious infections seen in 7% (28).

## JAK inhibitors in ASUC

Despite administration of infliximab or calcineurin inhibitors, patients being treated with these agents for rescue therapy remain at high risk for a colectomy. Studies show that rescue therapy still possesses a 20-30% rate of treatment failure on average, resulting in the need for a colectomy (62). As such, much work is being performed to identify therapeutics that may be used either consecutively to rescue therapy failure or in tangent to rescue therapies (**Table 1**). Recent work highlights the emergence of the use of Janus Kinase inhibitors (JAK). These agents work by inhibiting receptors found on intestinal epithelia that predominantly respond to inflammatory cytokines including interleukin (IL) 2,4,7,9,10,15 and 21 in addition to interferon (IFN) alpha and gamma which cause increased cellular permeability, damage and inflammation upon interaction with the JAK receptor (63). These agents function similarly to anti-TNF alpha agents such as IFX which inhibit the inflammatory agent from acting upon TNF receptors in addition to JAK receptors. Inhibition of the JAK receptor prevents downstream recruitment of cytosolic transcription factors Stat, thereby reducing the expression of genes mediating the inflammatory response (64). Due to their close interlinkage with regard to molecular pathway inhibition, JAK inhibitors could in theory be effective in the treatment of ASUC patients with IFX failure (65). Moreover, these agents could be used in combination to inhibit the same pathway from two directions and thereby potentially be more effective in achieving positive patient outcomes.

JAK inhibitors are attractive options for the treatment of ASUC due to their rapid gastrointestinal absorption and ability to induce swift clinical improvement as early as day 3 of treatment. Certain studies have highlighted JAK inhibitors’ pharmacodynamic superiority to other molecules due to decreased susceptibility to loss through the colonic mucosa owing to their non-protein-based formulation. Moreover, some studies suggest that JAK inhibitors may have a better safety profile only with regards to malignancy rates when compared to agents such as cyclosporine (66, 67). The most commonly studied JAK inhibitor is tofacitinib; however upadacitinib has also gained approval and popularity for the treatment of ASUC (61). Differences in these molecules lie in their breadth of molecular coverage. While tofacitinib is a pan-JAK inhibitor, upadacitinib selectively inhibits JAK1, thus lending to different efficacies and safety profiles. A recent study comparing these 2 agents head to head in UC reveals that upadacitinib is more efficacious in attainment of remission in moderate to severe UC (68). This finding is echoed in several other studies (30, 69, 70). This is theorized to be in part to the ability to dose upadacitinib at higher levels compared to tofacitinib, due to upadacitinib’s selectivity and avoidance of off target effects.

### Tofacitinib for induction of remission in ASUC

Singh et al. performed an RCT on the use of tofacitinib as an agent to induce remission in ASUC (34). The group assessed 78 patients over the age of 18 with moderately active UC. For a period of 8 weeks, patients were either administered oral prednisolone (40mg) four times daily with placebo or oral prednisolone (40mg) four times daily with tofacitinib (10mg) twice daily. Clinical remission was defined to be a total Mayo score ≤2 with endoscopic sub-score of 9 and fecal calprotectin <100ug/g and symptomatic remission was defined a normal stool frequency with absence of rectal bleeding. Achievement of clinical remission at week 8 was not significantly different between the two group, 16.28% of the tofacitinib group achieved remission while 8.57% of the prednisolone group achieved remission, (OR 2.07, 95% CI 0.49-8.70; p=0.31). Both groups achieved symptomatic remission at approximately 10 days. No serious adverse events occurred in either group. This data suggests further study in the use of tofacitinib as an induction agent, such that salvage therapies may not be necessary.

### Tofacitinib as salvage therapy for IFX failure

Tofacitinib is a small pan-JAK receptor inhibitor approved and well-studied for the treatment of ASUC. This agent is typically orally administered in 10mg doses up to two to three times a day. Additionally tofacitinib is unique in its ability to induce remission in as short as 3 days (31). While most studies dose patients on 10mg 3 times a day based off a phase 2 trial that highlighted efficacy of 30mg per day (71), tofacitinib usage to achieve remission appears to display a dose dependent pattern, with patients who receive the high dose of 15mg 2-3 times daily achieving higher rates of remission than those on 10mg (31, 72).

The OCTAVE studies outline the efficacy of this drug through the examination of 1139 patients who received either 10mg tofacitinib twice daily or placebo for 8 weeks and evaluated for daily mayo stool frequency and rectal bleeding. Measurements done at day 3 post initiation of treatment indicated that patients treated with tofacitinib had significant reductions from baseline stool frequency when compared to placebo (-0.27 vs. -0.11, p<0.01), number of daily bowel movements (-1.06 vs. -0.27, p<0.0001) and rectal bleeding as assessed by a sub score (-0.3 vs -0.14, p<0.01) (31). Similar results were seen the phase 4 TRIUMPH study in which 24 steroid refractory patients with ASUC were treated with tofacitinib 10mg twice a day and assessed for clinical response and biomarker improvement in 7 days. The mean baseline mayo score within patients was 10.1 (Standard deviation = 1.4). One third of these patients were anti-TNF agent refractory. Results indicated achievement of clinical response within 1 week in 58.3% of patients, with the mean number of days to achieve this being 2.4 days. Colectomy was only necessary in 16.7% of patients by day 7 and 25% of patients by month 6 (32).

A similar case series by Gilmore et al. examined 5 steroid and IFX refractory ASUC patients who all received high dose tofacitinib 10mg three times a day within 4 days of hospital admission and were followed for clinical response, remission and need for colectomy (73). Four out of the 5 patients demonstrated clinical response within the 4-day period of hospitalization. Patients who responded were continued at the same dose of tofacitinib for the first 2 weeks, after which the dose was reduced to 10mg twice a day for the subsequent 8 weeks. Patients who continued to respond were reduced to 5mg twice a day dosing. Follow-up on these 4 patients at 90 days showed maintenance of remission in all patients and a median mayo endoscopic score of 2. No adverse events were seen in the tofacitinib responders, with remission continued at month 7 of treatment (73). These results regarding both efficacy and safety were echoed in a case series which examined 12-month outcomes in 11 steroid and IFX refractory patients who were administered 3 doses of tofacitinib (10mg) daily. Ten of the 11 patients had clinical response to tofacitinib during initial hospitalization and 9 out of the 11 remained colectomy-free for 12 months (74). A case series by Berinstein et al. analyzed patients refractory to anti-TNF agents who were dosed on tofacitinib for 3 days (75) as opposed to the 2 week period seen in the previous study. Berinstein’s study evaluated 4 patients with ASUC who had failed IFX therapy and were determined to be likely to fail IV steroid monotherapy based on the Truelove and Witt’s criteria, inflammatory markers, endoscopic images, and prior medication failure. 3 of the 4 patients received tofacitinib 10mg 3 times a day while receiving IV methylprednisone 60mg daily for a period of 3 days. The fourth patient was given budesonide alongside tofacitinib due to prior exacerbation of psychiatric illness with corticosteroids. All 4 patients had rapid improvement in symptoms and decline in CRP by hospital day 5, with only 1 patient (dosed on methylprednisone and tofacitinib) not achieving clinical remission. It must be noted that the patient that did not achieve remission had the highest baseline CRP level and colonic dilation. However, 2 out the 4 patients elected to undergo colectomy done at 6 months due to concerns for multifocal dysplasia. At follow up of the patients at 18 months, there were no major adverse events, with only one patient (administered both methylprednisone and tofacitinib) developing a nonspecific truncal maculopapular rash (75).

For a particular case of steroid refractory and IFX non-responsive ASUC, tofacitinib used in conjunction with cyclosporine was found to be effective in the induction of remission (33). The patient was administered IV cyclosporine (3mg/kg/day) along with oral tofacitinib (10mg twice daily). By day 10 of therapy, the patient noted complete resolution of abdominal pain and a decrease in the number of stools per day from over 10 to less than 3, without any signs of bleeding. At 1 month, the treatment plan was modified to oral cyclosporine (150mg, twice daily) with tofacitinib (5mg, twice daily). The patient’s condition remained stable after 6 months when cyclosporine was discontinued and she was given tofacitinib (5mg, twice daily) with mesalazine (1.5g, twice daily). After 1 year, tofacitinib was discontinued. Endoscopic evaluation revealed intestinal mucosal ulcer healing with scarring.

The provided data makes tofacitinib a potentially attractive agent for the salvage of IFX refractory ASUC patients. A systematic review by Steenholdt et al. analyzed 21 studies comprising 148 cases of steroid and IFX refractory ASUC patients, to examine the efficacy of this therapy. Tofacitinib provided 85%, 86% and 69% of patients with 30-day, 90 day and 180-day colectomy free survival, respectively, with standard dosing regimens. Persistence of remission after 180 days post initiation of therapy ranged between 68-91% and endoscopic remission was seen in 55% of patients. However, 22 patients faced severe adverse effects of which 13 were due to herpes zoster infections (29).

While there is encouraging data regarding the potential usage of tofacitinib as a salvage therapy for IFX and steroid refractory ASUC patients, further prospective studies are warranted to fully elucidate efficacy and safety of this sequence and additionally derive optimal dosing regimens.

### Upadacitinib as salvage therapy for IFX failure

Upadacitinib is a JAK 1 inhibitor, gaining popularity for the salvage therapy of IFX and steroid failure in ASUC patients. Its rapid onset of action allows for attainment of clinical reduction in symptoms within 1 day of usage, making it a compelling potential agent as salvage therapy (30).

Given the more recent introduction of upadacitinib in IBD treatment, robust data to evaluate its usage is lacking, with some studies looking at its use when co-administered with corticosteroids. Berinstein et al. studied its efficacy when co-administered with IV corticosteroids in 25 ASUC patients (76). 24% of patients underwent colectomy within 90 days of treatment initiation of which some were elective, while 83% maintained remission. Only 1 patient experienced a venous thromboembolic adverse event (76). Further prospective studies would be necessary to inform its usage.

On the other hand, sequential administration of upadacitinib after IFX and steroid failure in ASUC patients was reported in a study by Gilmore et al. (77). This group identified 6 patients at 2 Australian tertiary inflammatory bowel disease centers who were administered 45mg of upadacitinib daily whist transitioning IV corticosteroids to oral corticosteroids on day 3 of hospital admission and followed them for 16 weeks post discharge. All patients were previous exposed to IFX. On day 5 of hospital admission, 5 of the 6 patients demonstrated clinical response as defined by the modified Oxford criteria with the 6^th^ patient achieving response at day 7. By week 8, 5 patients were able to wean off oral corticosteroids, with 4 of the patients maintaining clinical remission. At the same time point, endoscopic examination revealed that 50% of patients achieved remission with a median bowel wall thickness of 2 mm. After week 8, 67% of patients remained on 45mg daily dosing, while the remaining patients were able to decrease their dose to 30mg maintenance even by week 8. Only one patient needed colectomy at day 16 due to ongoing severe disease activity (77). Similar results with regards to efficacy were seen in a case series by Zinger et al. (78). 4 patients with IFX and steroid refractory ASUC with a baseline endoscopic mayo score of 3 were administered 45mg/day upadacitinib. 3 of the 4 patients achieved clinical response within 4 to 8 days of treatment initiation. Follow up at 3 months post hospitalization revealed that 2 patients were able to be weaned off steroids and remain in steroid free clinical and endoscopic remission. The third patient maintained clinical response without achieving remission, and the fourth patient had to undergo total colectomy.

Damianos et al. recently conducted a systematic review assessing use of upadacitinib for ASUC. The review included 11 studies with a total of 55 patients and found that most patients experienced rapid induction of remission and sustained remission thereafter. Colectomy rates were found to be 16.3% at 90 days following initiation of upadacitinib. 80% of patients who did not undergo colectomy maintained long term steroid-free remission. Additionally, the rate of adverse events was low, with only 2 thromboembolic events seen (79).

## Future directions

ASUC remains a serious medical concern in the realm of IBD. Due to its severity and life-threatening nature, prompt evaluation and initiation of treatment is required. While the majority of currently available treatments are successful in achieving short-term remission, patients remain at high risk for requiring a future colectomy. As such, it is critical to develop methods for early identification of patients who would be good candidates for medical therapy and, moreover, identify medical therapies with efficacy, tolerability, and safety in long term use.

Fast-acting biologic therapies are good candidates as agents that may be used long term due to efficacy and ease of administration. Current investigations into developing protocols for individualized dosing to achieve predicted trough levels of infliximab may help optimize patient outcomes. However, given the need for colectomy in a significant proportion of patients treated with infliximab, it is necessary to identify good second line salvage agents when biologic therapies fail.

Novel research on JAK inhibitors for salvage are promising, but further prospective studies are needed to evaluate efficacy of these drugs. Future studies are also required to evaluate the safety profile of these rescue therapies and predict which patients may be at high risk of developing adverse events. Current potential issues with regards to these therapeutics are risks of thromboembolic events, neutropenia and zoster (80). Future prospective trials may aim to identify baseline patient characteristics that would predispose patients to adverse events, particularly with respect to JAK inhibitors. With more specific trials evaluating this population, the algorithm for the treatment of patients with ASUC may be optimized to achieve improved patient outcomes.
